# Open-Source Browser-Based Tools for Structure-Based Computer-Aided Drug Discovery

**DOI:** 10.3390/molecules27144623

**Published:** 2022-07-20

**Authors:** Ann Wang, Jacob D. Durrant

**Affiliations:** Department of Biological Sciences, University of Pittsburgh, Pittsburgh, PA 15260, USA; anw152@pitt.edu

**Keywords:** computer docking, computer-aided drug discovery, tool development, open source, usability, web-based tools, browser-based tools

## Abstract

We here outline the importance of open-source, accessible tools for computer-aided drug discovery (CADD). We begin with a discussion of drug discovery in general to provide context for a subsequent discussion of structure-based CADD applied to small-molecule ligand discovery. Next, we identify usability challenges common to many open-source CADD tools. To address these challenges, we propose a browser-based approach to CADD tool deployment in which CADD calculations run in modern web browsers on users’ local computers. The browser app approach eliminates the need for user-initiated download and installation, ensures broad operating system compatibility, enables easy updates, and provides a user-friendly graphical user interface. Unlike server apps—which run calculations “in the cloud” rather than on users’ local computers—browser apps do not require users to upload proprietary information to a third-party (remote) server. They also eliminate the need for the difficult-to-maintain computer infrastructure required to run user-initiated calculations remotely. We conclude by describing some CADD browser apps developed in our lab, which illustrate the utility of this approach. Aside from introducing readers to these specific tools, we are hopeful that this review highlights the need for additional browser-compatible, user-friendly CADD software.

## 1. Introduction

This review describes the importance of open-source, accessible tools for structure-based computer-aided drug discovery (CADD). To provide context, the article begins with a summary of drug discovery in general and CADD in particular. A discussion of software usability follows, focused on the shortcomings of common software deployment approaches, as well as possible solutions. Finally, we use several of our group’s own research tools to illustrate potential software development methods that balance utility and usability.

### 1.1. Drug Classifications: Biologics and Small Molecules

Pharmaceutical drugs are agents that improve health by modulating the activity of disease-implicated macromolecular targets such as proteins. They can be broadly categorized as biologics—substances produced by living organisms (e.g., antibodies and interleukins) [1,2]—and small-molecule (synthetic) compounds. This review focuses on the latter, but we certainly acknowledge the critical role that computation has also played in furthering the design of biologics. Biologics have many advantages over small molecules. For example, they can potentially target disease-implicated proteins whose activity depends on interactions with other protein partners via flat surfaces. Many biologics also benefit from high affinity and specificity, which reduces off-target toxicity. However, notable disadvantages include complex and expensive manufacturing processes, vulnerability to degradation and microbial contamination, the potential for adverse immune responses, invasive routes of administration (e.g., injection), poor pharmacokinetic properties (e.g., limited distribution), and higher patient costs [3,4].

In contrast, small-molecule drugs are low-molecular-weight chemical compounds that bind in pockets on the surfaces of disease-implicated proteins. Such drugs have several advantages over biologics [3,4], including increased membrane permeability in some cases, the potential for simplified (e.g., oral) administration, comparatively straightforward and more scalable manufacturing, reduced immunogenicity, and often reduced patient cost. However, developing small molecules with high affinity and specificity is challenging and requires extensive lead optimization. While poor specificity can be beneficial in some cases (e.g., polypharmacology), off-target binding typically leads to undesirable side effects. Additionally, small-molecule drug targets are almost exclusively limited to those with well-defined binding pockets [5].

### 1.2. Computer-Aided Drug Discovery

Most clinically approved drugs are small molecules [4,5], but designing these drugs continues to be costly. Recent estimates suggest that it typically takes over a decade of development [6]—and roughly a billion dollars [7]—to bring a new drug to the market. Computer-aided drug discovery (CADD) is a popular approach to expediting the process. So-called ligand-based CADD leverages information about known small-molecule binders to predict which additional molecules might also be pharmacologically active. By considering the physicochemical and structural properties of known bioactive compounds, one can design related molecules with improved affinities or other properties, even when the specific target is unknown.

However, ligand-based methods have several drawbacks. For example, they may fail to identify novel ligands that are substantially different from the known “prior art”, and they are ineffective against targets with no known ligands—arguably the most interesting from a pharmacological perspective. The assumption that minor chemical modifications lead to incremental changes in pharmacological potential (e.g., potency) is also flawed in many cases. Medicinal chemists often observe activity cliffs when testing congeneric series of analogs, wherein slight chemical changes yield large differences in potency. Such cliffs invalidate the linearity often assumed in predictive ligand-based structure–activity relationship (SAR) analyses [8].

In contrast, receptor-based (i.e., structure-based) CADD leverages the three-dimensional structure of a known target (e.g., a disease-implicated protein) to identify new ligands. Receptor-based CADD does not depend on already known ligands and so can identify first-in-class bioactive molecules. However, structure-based approaches require a known drug target with a known, atomic-resolution structure. 

## 2. Software Usability 

Having discussed drug classifications and discovery strategies, we now describe the usability challenges associated with many otherwise powerful and effective CADD tools. Usability is not simply a convenience; it can drastically impact adoption. The best programs are both accurate in their predictions and easy to use.

### 2.1. Common Usability Challenges

Downloading and installing software seems trivial, but it presents a small barrier with an outsized impact on adoption. Although downloading from the internet is straightforward for most users, many programs are distributed through command-line package managers (e.g., *apt-get*, *yum*, *npm*, and *pip*). These package managers are difficult for novices to use, and many programs have dependencies (e.g., Python packages) that require additional downloads. If software is distributed as source code, compiling and installing the final product can also be challenging, often requiring extensive configuration. Finally, many programs do not auto-update, so users must repeat the download/installation process each time a new version is released.

CADD tools often only run in specific environments, further complicating use. For example, some require a specific operating system (e.g., Linux but not Windows or iOS), others require a particular version of a third-party library (e.g., NumPy 1.11 but not NumPy 1.12 or later), and still others require a specific programming-language interpreter (e.g., Python2 but not Python3). Users accustomed to one environment may not be able to use programs that require a different environment.

Finally, many tools lack graphical user interfaces (GUIs), requiring users to enter commands into a command-line terminal (i.e., a text-based, UNIX- or MS-DOS-like environment). Such command-line interfaces (CLIs) are useful when advanced users wish to automate analyses via scripting. CLIs are also ideal when running software via a remote terminal (e.g., SSH) that lacks a graphical desktop environment—as is common in high-performance computing. However, GUI-based tools are arguably better suited for the broader scientific community.

### 2.2. Server Applications

The “server app” software deployment model improves usability by allowing users to simply visit a web page where they can upload their local data to a remote server. The required calculations are then performed “in the cloud”. When finished, the server sends the results back to the browser so that the user can save them locally. This approach does not require users to download and install software; is accessible from any operating system with a modern web browser (including mobile); gives the programmer (rather than the end user) control over the environment where the calculations run; enables software updates server-side without requiring end-user intervention; and provides an easy-to-use, browser-based GUI.

However, the server-app approach has some notable disadvantages. Users must upload their (possibly proprietary) data to a third-party server and trust that the data will be kept private and safeguarded from data breaches. If remote resources are limited, the server app may be forced to implement a queue system, which can delay start times. The server-app approach also prevents other programmers from easily incorporating the remote functionality into their own applications and workflows, unless the remote resource provides an application programming interface (API). Finally, users must trust that the server will be reliably available. Maintaining substantial remote resources requires both staffing and funding; if staff members change jobs or grant funds run out, critical components of an effective CADD pipeline might be suddenly and permanently taken offline.

### 2.3. Browser Applications

In contrast, the “browser app” software deployment model turns the “server app” model on its head. Browser-app-enabled web pages do not require users to upload their data to the cloud; instead, the remote server sends the required analysis software to the user’s local browser, where the calculations occur [9,10]. Given that these apps depend on locally available compute resources, they are not well suited for high-performance-computing calculations (e.g., molecular dynamics simulations of whole proteins) or calculations that require local access to large databases (e.g., homology modeling with AlphaFold2 [11]). However, many common CADD tasks are far less demanding and can efficiently run in a browser.

Browser apps retain many of the advantages of server apps. For example, the analysis software is automatically transferred to users’ local web browsers when they visit a browser app web page, so no direct download or installation is required. Browser apps are also accessible from all major operating systems because all such systems have modern web browsers (e.g., Google Chrome, Firefox, Safari, and Edge). These browsers provide a standard computing environment that is the same everywhere, so developers do not need to accommodate different operating systems explicitly. Updating the software is as easy as updating a web page (requiring no additional end-user action). Finally, users can control browser apps from easy-to-use HTML5/JavaScript GUIs. 

Browser apps also overcome many of the disadvantages typical of server apps. The calculations occur in the local web browser, so uploading user data to a third-party server is never necessary. Moreover, each user provides the required compute resources to run his or her calculations, eliminating the need for “cloud-based” computing infrastructure and queue systems. Indeed, hosting a browser app is no more difficult than hosting a standard web page. Finally, a browser app’s core functionality can be packaged into a library that other programmers can incorporate into their projects without requiring direct access to a remote server/resource.

### 2.4. Recent Advances Enable Complex Browser-Based Applications

Historically, developing complex applications that run in a web browser has been prohibitively challenging. Browsers use the JavaScript programming language, which lacks many advanced features typical of languages such as C and C++. Several recent developments have mitigated this challenge. First, much work has focused on creating tools that can translate (“transpile”) source code written in feature-rich languages into JavaScript [9]. For example, the TypeScript [12] programming language has JavaScript-like syntax but provides features that vanilla JavaScript lacks (e.g., optional static typing, classes, and interfaces). Once transpiled to JavaScript, TypeScript code runs seamlessly in a browser. A subset of the Python programming language, which plays a prominent role in CADD software development, can also be transpiled to JavaScript using tools such as Transcrypt [13] and Brython [14].

Second, WebAssembly [9,15,16,17] has made it easier to run complex applications in the browser environment. WebAssembly allows programmers to compile computer code written in languages such as C, C++, and Rust to a binary format (unrelated to JavaScript) that runs in any modern web browser, just as one might compile code to run on Windows, Linux, or macOS. The browser provides an operating-system-independent environment (“virtual machine”) to run the software. Several popular CADD libraries and programs have been successfully compiled to WebAssembly, including software for pocket identification [18], computer docking [19], chemical file conversion [16,17], cheminformatics analysis [9,17], and molecular simulation [17]. One can even use WebAssembly to run scripts written in interpreted (non-compiled) languages if the associated interpreter is compiled to WebAssembly. For example, Pyodide [20], a WebAssembly-compiled version of the CPython interpreter, runs Python scripts and even provides access to libraries such as NumPy [21], SciPy [22], and Biopython [23].

Third, recent JavaScript APIs enable access to host-computer hardware that was previously inaccessible, greatly expanding JavaScript’s functionality [9,24]. Notable browser APIs include WebGL and WebGPU, which enable GPU-accelerated graphics and calculations, and WebXR, which provides access to virtual and augmented reality headsets. The web community has built on these APIs, creating open-source JavaScript libraries capable of even more sophisticated tasks. For example, the Babylon.js library [25] leverages WebGL, WebGPU, and WebXR to provide a full-fledged 3D game engine that runs in a web browser. And the TensorFlow.js library [26] uses WebGL and WebGPU to enable the training and inference of complex machine-learning models.

Given these advances, it is now possible to build and run many components of a typical CADD pipeline in a browser environment. The remainder of this review describes our efforts to implement some of these components as easily accessed browser apps.

## 3. Examples of CADD Browser Apps

### 3.1. FPocketWeb: Pocket Identification

Small-molecule drug discovery aims to find chemical compounds that bind in pockets on macromolecular (e.g., protein) surfaces. Often, the location of a candidate pocket is unambiguous. For example, perhaps the protein has been cocrystallized with a bound ligand, homologous proteins provide insight into the pocket location, or mutagenesis studies have identified binding-implicated residues. However, many binding pockets are uncharacterized, especially when a drug target has no known ligands or possesses uncharacterized allosteric sites.

Several computational tools aim to identify binding pockets based on the structure of the drug target (e.g., FINDSITE [27], COACH [28], and SITEHOUND [29]; reviewed recently in Ref. [30]). Among these programs, *fpocket* is particularly popular [31]. *fpocket* accepts a receptor PDB file as input and returns a ranked list of potential binding pockets identified using a sphere-based approach. First, *fpocket* lines the protein surface with so-called “alpha” spheres using Voronoi tessellation. It then identifies clusters of spheres as candidate pockets. Finally, it ranks each candidate pocket by its predicted ability to bind small molecules. Of note, *fpocket* has been recently used in projects focused on druggable SARS-CoV-2 RNA structural elements [32], AT1-receptor allosteric sites [33], and Hv1-channel inhibitors [34]. 

Though powerful, *fpocket* is a CLI tool. To improve usability, the *fpocket* creators implemented the algorithm as a server application, making it as easy to use as visiting a website. This focus on usability is laudable and serves the needs of many users. However, in some cases, the limitations of server apps described above may give some users pause. Moreover, because other programmers are (understandably) denied direct access to the *fpocket* server, they cannot easily incorporate this server-based implementation into their own applications and workflows.

To build on this past work, we created FPocketWeb, a browser app implementation of *fpocket3*. FPocketWeb consists of two components: (1) a browser-based library that implements the CADD tool itself and (2) a GUI that allows the user to configure the tool, run it in the browser, and visualize the output. To create the FPocketWeb library, we used the Emscripten toolchain [35] to compile *fpocket3* [36] to WebAssembly. The compiled FPocketWeb library is available from our website (Table 1) and can be freely incorporated into other browser-based projects.

**Figure 1:**
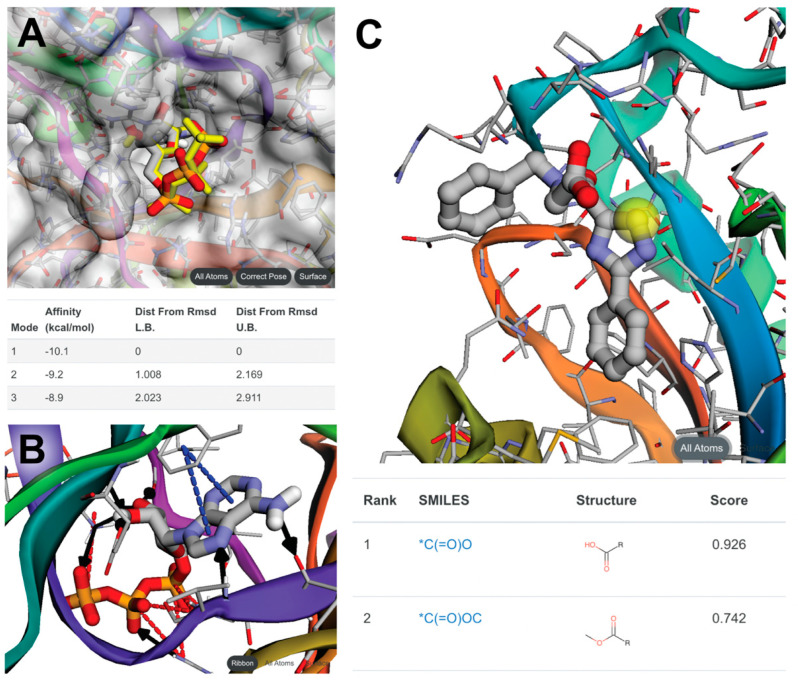
Three examples of browser app output. All three examples demonstrate how molecular visualization is a critical component of our in-browser approach. (**A**) Webina outputs predicted ligand poses and scores. (**B**) BINANA represents identified protein/ligand interactions as solid and dashed lines. (**C**) DeepFrag shows the protein, ligand, and ligand atom to which optimizing fragments should be added (yellow sphere). The recommended fragment additions are displayed below.

To create the FPocketWeb GUI, we used the TypeScript programming language and the open-source Vue.js framework [37]. Vue.js allows programmers to create reusable components (e.g., buttons and text fields), ensuring a user interface consistency that also contributes to usability. We styled these components according to the Bootstrap framework [38] originally developed at Twitter. Incorporating Bootstrap styling into a Vue.js app is straightforward thanks to the open-source BootstrapVue [39] library. Finally, given that in-browser molecular visualization is critical for many of our browser apps, we developed a Vue.js component based on the popular 3Dmol.js visualization library [40] (Figure 1). 

Once we finalized the FPocketWeb library and GUI, we compiled, assembled, and optimized the components using Webpack [41] and Google’s Closure Compiler [42] to produce the final browser app. The app and source code are available online under the open-source Apache License, version 2.0 (Table 1). We have published full details regarding FPocketWeb use, performance, and benchmarking on bioRxiv [18] and intend to publish a related peer-reviewed manuscript soon.

### 3.2. Webina: Small-Molecule Docking

After one identifies the location of a potential binding pocket, a natural next step is to identify drug-like small molecules that might bind in that pocket. Docking programs leverage protein and small-molecule structures to predict binding. They first position virtual small molecules within a specified binding pocket. The geometry of the bound molecule relative to the target is called the predicted pose. Second, they map that pose to some score that (hopefully) correlates with affinity. Ranking compounds by their docking scores allows one to prioritize top-ranked compounds for subsequent experimental evaluation.

Several powerful docking programs are free for academic use, and some are released under even less restrictive licenses [43]. Examples of these include AutoDock 4 [44], AutoDock Vina [45,46], UCSF DOCK [47], FLIPDock [48], EADock [49,50], and PatchDock [51]. AutoDock Vina [45,46] (Vina) is particularly popular because of its reasonable accuracy and straightforward use. As input, Vina accepts models of the protein receptor and candidate small-molecule ligand in the PDBQT format, as well as the location and size of a docking box that encompasses the binding pocket of interest. As output, Vina produces a list of candidate ligand poses with associated docking scores.

Although several programs (requiring separate download, installation, and use) serve as GUI wrappers around the Vina executable (e.g., AMDock [52], PyRx [53], AUDocker LE [54], DockoMatic [55], the PyMOL AutoDock plugin [56], and DockingApp [57]), Vina itself uses a CLI. The CLI approach is particularly challenging in this context, given that proper Vina use depends on molecular visualization, which is only available through third-party viewers [44,58,59,60,61]. For example, visualization is critical when defining the pocket-encompassing docking box. The box must not be so small that it excludes some portions of the pocket, but it must not be so large that the subsequent conformational search is prohibitively expensive. Molecular visualization is also essential for analyzing Vina’s output, which includes predicted ligand poses. 

To address these challenges, we compiled the Vina codebase (version 1.1.2) to WebAssembly. Using the same approach used to create FPocketWeb, we integrated the compiled library into a GUI-based browser app called Webina [19]. Aside from running Vina in the browser, Webina allows users to specify the docking box via our 3Dmol.js-powered molecular viewer component. Once the calculation finishes, Webina displays the predicted ligand poses and associated scores in the browser without requiring a third-party molecular visualization program (Figure 1A). Webina thus simplifies the docking process, making it accessible even to novices. Though only recently published, several researchers have already used Webina to study compounds with analgesic [62], anti-cancer [63,64], anti-bacterial [65], anti-viral [66,67,68], and antioxidant [69] properties, among others [70,71]. The Webina app and source code are freely available online under the terms of the open-source Apache License, version 2.0 (Table 1). 

### 3.3. BINANA: Pose Assessment

After one identifies a predicted ligand pose, a natural next step is to characterize and visualize the interactions that the candidate ligand may form with the drug-target receptor (e.g., hydrogen, hydrophobic, salt-bridge, and van der Waals interactions). Understanding these interactions can provide insight into the target protein’s mechanism of action. It is also useful for assessing the pharmacological potential of small-molecule drug candidates and so can guide decision making during the development process. 

Several popular desktop molecular visualization programs [58,59,61] can identify protein/ligand interactions, but users cannot access these tools through a browser-based interface and so must download and install them on their local machines. Some of these programs are also free only for noncommercial use [58,61]. Recognizing the importance of easy access, others have created server apps to characterize protein/ligand interactions (e.g., Arpeggio [72] and PLIP [73]); though laudably accessible, these tools are copyleft licensed, which may also limit commercial use. Furthermore, the server-app approach makes it difficult for other programmers to easily incorporate the third-party functionality into their own programs and workflows.

To address these issues, we recently modernized the BINding ANAlyzer (BINANA) program [74,75], which aims to improve the efficiency of ligand-binding characterization by automating ligand-pose analyses. Specifically, we updated the Python codebase and created a JavaScript library to enable analysis in the browser. To generate the JavaScript version, we used Transcrypt [13] to translate (or “transpile”) the BINANA Python code directly into JavaScript. Others are free to use this library in their browser-based projects; to demonstrate, we integrated it into a browser app created using the same TypeScript/Vue.js approach described above.

BINANA accepts the structures of a drug target and bound small molecule as input. Considering the locations and orientations of the chemical groups on both, it predicts hydrophobic, salt-bridge, π–π, T-stacking, cation−π, hydrogen-bond, halogen-bond, and metal-coordination interactions. The browser app displays the interactions without requiring a third-party visualization program (Figure 1B). The app and source code are available online under the terms of the open-source Apache License, version 2.0 (Table 1). 

### 3.4. DeepFrag: Lead Optimization

Small-molecule ligands identified through virtual and ex silico screening rarely have the binding kinetics typical of FDA-approved drugs. Hit-to-lead optimization is the process by which an initial “hit” (i.e., a molecule that interacts with a drug target even if only weakly) is transformed via molecular fragment additions or replacements into a “lead” (i.e., a compound with improved potency, selectivity, or other pharmacokinetic/toxicological properties [76]). Further lead optimization similarly transforms leads into improved compounds ready for preclinical assessment [77].

Identifying chemical modifications that improve drug-relevant properties is rarely straightforward. Several existing machine-learning approaches serve as structure-based hypothesis-generation tools to assist with hit-to-lead and lead optimization. These tools can be broadly divided into ligand-based and structure-based approaches [78]. Ligand-based techniques leverage known ligands to predict optimization strategies without regard for the structure of the target binding pocket. Examples of these include Mol-CycleGAN [79], JT-VAE [80], GENTRL [81], CGVAE [82], and MolDQN [83], among others [84,85,86,87,88]. In contrast, structure-based approaches leverage 3D structural information (e.g., crystallographic, NMR, or modeled receptor structures) to suggest optimization strategies. Examples of these include DeepLigBuilder [89], DEVELOP [90], and 3D-Scaffold [91], among others [76].

Building on this previous work, we created a deep convolutional neural network capable of recommending optimizing fragment additions. Our DeepFrag model [92,93] uses a structure-based approach; as input, it takes the 3D structure of a protein drug target, the 3D structure of a posed (bound) ligand, and the 3D coordinates of a ligand atom to which some optimizing molecular fragment should be added. DeepFrag voxelizes the receptor and ligand by projecting them onto a 3D grid. It then applies a series of (primarily) 3D convolutional layers to the voxelized images. The last convolution is flattened and eventually fed into a fully connected neural network whose output is an RDKFingerprint-like vector [94] of floating-point numbers that describes the topological features of the predicted optimizing fragments. To find the structures of suitably similar fragment matches, one can compare the DeepFrag-predicted fingerprint to the pre-computed fingerprints of many known fragments in a molecular library. To the best of our knowledge, DeepFrag is the first machine-learning approach that formulates lead optimization as a classification problem (rather than a generative-modeling problem) by predicting fragment fingerprints from 3D voxel representations. 

DeepFrag was originally implemented in Python and designed for use via a CLI. To encourage broad adoption, we converted the trained model to a format compatible with the TensorFlow.js JavaScript library, which enables deep learning in the browser. TensorFlow.js relies on several browser technologies, including WebAssembly and WebGL, to perform the required computations quickly. Using TensorFlow.js, others can incorporate our DeepFrag model into their browser apps.

To demonstrate, we created a browser app that incorporates the DeepFrag model [92]. We used the same TypeScript/Vue.js approach described above to create the GUI. The app also performs the fingerprint-matching step of the DeepFrag workflow, returning the actual structures (rather than fingerprints) of suitable fragments for scaffold addition (Figure 1C). The app and source code are available online under the open-source Apache License, version 2.0 (Table 1).

### 3.5. ProteinVR: Molecular Visualization in Virtual Reality

The importance of molecular visualization in any CADD pipeline cannot be overstated. To fully understand how a small-molecule ligand might bind to a protein target, one must fully appreciate the spatial relationships between the ligand’s chemical moieties and the protein’s amino acids. This understanding also provides valuable insights that can guide lead optimization.

Existing molecular visualization programs include VMD [58], PyMOL [59], UCSF Chimera [61], and ChimeraX [60]. These programs primarily convey structural information by projecting 3D molecular models onto 2D screens. Rotating the molecular structures or using simulated fog can convey some three-dimensional information. However, it is difficult to immediately and fully intuit protein/ligand interactions and other spatial elements using this approach. Molecular visualization in virtual reality (VR) helps overcome this challenge. Such visualization grows in popularity as the price of VR headsets declines. Indeed, one can purchase a standalone VR headset for under USD 300, and the price will likely continue to drop. 

Most VR molecular visualization programs run as dedicated desktop applications [60,95,96,97]. The desktop approach is ideal in many cases because it enables innovative navigation methods [95], resource-intensive molecular-editing tools [97], and real-time user interactions with ongoing molecular dynamics simulations [96,98,99,100,101,102,103]. However, many situations call for quick, easily accessible VR visualization, and desktop programs require download, installation, and experience to use effectively. Additionally, many desktop programs only support high-end VR devices [60,95], and some require a commercial license to enable anything beyond the most basic functionality [97].

To further advance the community’s interest in VR applied to molecular visualization, we created the ProteinVR browser app [104]. ProteinVR provides many of the same molecular insights as desktop VR programs. However, it delivers those insights via a web browser, bypassing the need for separate download and installation. Users simply load molecular structures into their browsers’ memory, either from a file on their computer or by automatically interfacing with online resources (e.g., the PDB). Once a file is loaded, users can modify the visualization (e.g., which color scheme to use; whether to represent proteins as ribbons, surfaces, etc.; whether to represent small molecules as sticks, spheres, etc.). They can also easily share molecular scenes by simply sending custom URLs to colleagues.

ProteinVR is built using the Babylon.js JavaScript library [25], a full-featured browser-based game engine that we repurposed for molecular visualization. Implementing a game engine in the browser is only possible because of recent JavaScript APIs that improve access to host-computer hardware. Babylon.js specifically leverages WebGL for browser-based 3D graphics and WebXR to support browser-based virtual reality on a broad range of VR headsets.

A freely accessible ProteinVR implementation and the app’s source code are available online under the open-source 3-Clause BSD License (Table 1).

## 4. Browser Apps as Educational Tools

The CADD browser apps highlighted in this review were designed primarily as research tools, but the emphasis on easy access and usability also makes them well suited to educational settings. Indeed, the corresponding author has successfully used some of these tools (Webina, ProteinVR, and DeepFrag) in the classroom and has received positive feedback from other educators. 

Browser apps are valuable tools for incorporating active-learning exercises into the classroom [105]. Active learning encourages students to actively participate in the learning process, beyond just passive listening. It promotes learning by engaging students in real-world problem solving [106]. Such exercises are particularly useful for new computational biology/chemistry students; scientific computation is foreign to many of them, so even small barriers can limit the benefit of CADD-focused active-learning exercises.

The first common barrier is accessibility. Some undergraduate classes have dozens or even hundreds of students. Expecting so many students to separately download and install a CADD tool that may not even be compatible with their operating system is impractical. Yet nearly all students know how to visit a web page, and browser apps work seamlessly on all major operating systems. These apps can thus introduce students to advanced computational tools that they could not otherwise access.

The second barrier is usability. While advanced undergraduates may be familiar with CLIs, younger students often are not. Active-learning projects using CLI CADD tools require students to not only understand the tool itself but also the non-intuitive command-line interface required to run that tool. In contrast, browser apps provide easy-to-use GUIs that students can launch by simply visiting a URL, allowing them to focus on their results rather than on usability hurdles.

The third barrier is technical. In large classroom settings, active-learning activities often require many students to use the same tool simultaneously. If these activities leverage server apps, the many simultaneous requests can quickly overwhelm the remote resource. The remote server must often implement lengthy wait times to deal with the sudden demand, and such delays are not conducive to student learning. In contrast, browser apps perform the calculations on each student’s own computer and so are less likely to be overwhelmed at moments of high demand.

Finally, socioeconomic barriers also complicate CADD-focused active learning. Studies suggest students from challenging socio-economic backgrounds tend to select universities closer to their homes [107,108], but many live far from universities with the shared infrastructure required to support a computationally oriented curriculum. Browser apps distribute the computations to each student’s personal device rather than requiring a shared resource. They thus have potential to democratize computational chemical biology education.

## 5. Conclusions

Many powerful CADD tools accelerate early-stage drug discovery. Though broadly adopted, these tools do not always provide an easy-to-use interface that can enable even greater adoption. Our group found that browser apps are well-suited for CADD tool deployment. A simple web server sends a CADD analysis program to the user’s local browser when they first visit the app webpage, thus eliminating the need for manual download and installation. Calculations take place on the user’s local computer rather than on a third-party resource, so the user never needs to send proprietary data to a remote system. All major operating systems have modern browsers, so browser apps are broadly compatible by design. Moreover, thanks to HTML, JavaScript, and other tools, one can easily create user-friendly GUIs to set up calculations and visualize results.

As JavaScript and related web technologies advance, we anticipate that browsers will become increasingly powerful platforms for software deployment. This migration to the browser is already apparent in other areas; for example, both Google and Microsoft have developed web-based word processors, spreadsheets, and presentation applications with substantial browser-side components. Given that web browsers are ubiquitous, operate across multiple platforms, and are well suited to visualization, we anticipate that CADD tools will increasingly leverage the browser as a software deployment platform.

## Figures and Tables

**Table 1:** Examples of CADD browser apps created in the Durrant lab.

Name	App URL ^1^	Source Code URL ^1^	License/Method ^2^	Step
FPocketWeb	/fpocketweb	/fpocketweb-download	AL2/Wasm	Pocket
Webina	/webina	/webina-download	AL2/Wasm	Dock
BINANA	/binana	/binana-download	AL2/Transcrypt	Assess
DeepFrag	/deepfrag	/deepfragmodel	AL2/TF.js	Optimize
ProteinVR	/pvr	/protein-vr	BSD3/Babylon.js	Visualize

^1^ All URLs are relative to durrantlab.com (e.g., http://durrantlab.com/fpocketweb). ^2^ “AL2” stands for the Apache License, version 2.0; “BSD3” stands for the 3-Clause BSD License; “Wasm” stands for WebAssembly; “TF.js” stands for TensforFlow.js.

## Data Availability

Not applicable.
